# HIPK2 Overexpression and Its Prognostic Role in Human Papillomavirus-Positive Tonsillar Squamous Cell Carcinoma

**DOI:** 10.1155/2017/1056427

**Published:** 2017-05-21

**Authors:** Mi Jung Kwon, So Young Kang, Eun Sook Nam, Seong Jin Cho, Young-Soo Rho

**Affiliations:** ^1^Department of Pathology, Hallym University Sacred Heart Hospital, Hallym University College of Medicine, Anyang, Gyeonggi-do 431-070, Republic of Korea; ^2^Department of Pathology, Samsung Medical Center, Sungkyunkwan University College of Medicine, Seoul 135-710, Republic of Korea; ^3^Department of Pathology, Kangdong Sacred Heart Hospital, Hallym University College of Medicine, Seoul 134-701, Republic of Korea; ^4^Department of Otorhinolaryngology-Head and Neck Surgery, Ilsong Memorial Institute Head and Neck Cancer Center, Kangdong Sacred Heart Hospital, Hallym University College of Medicine, Seoul, Republic of Korea

## Abstract

Tonsillar squamous cell carcinomas (TSCCs) are the most common human papillomavirus- (HPV-) associated oropharyngeal cancers with poor prognosis. Homeodomain-interacting protein kinase 2 (HIPK2) is a central regulator of p53, which participates in apoptosis during the DNA damage response. HIPK2 is involved in HPV-associated uterine cervical and cutaneous carcinogenesis through its binding of HPV E6, thereby preventing apoptosis and contributing to tumor progression. However, its clinical and prognostic significance in TSCC remains unclear.* HIPK2* mRNA levels were analyzed in 20 normal tonsils and 20 TSCC specimens using real-time reverse transcription polymerase chain reaction. Immunohistochemistry of HIPK2 was performed in 79 resected specimens. HIPK2 was expressed in 57% of the TSCCs, and HIPK2 protein expression and* HIPK2* mRNA levels were higher in TSCCs than in normal tonsils. HIPK2 overexpression was associated with poorly differentiated carcinoma and low alcohol consumption and was an independent prognostic factor for overall survival and disease-free survival (DFS) in TSCC and a negative independent prognostic factor for DFS in patients receiving postoperative radiotherapy. HIPK2 overexpression had a significant association with poorer DFS in HPV-positive TSCCs, but not in HPV-negative tumors. HIPK2 overexpression may be a potential prognostic marker for predicting prognoses and a high risk of recurrence, particularly in patients with HPV-positive TSCC.

## 1. Introduction

Tonsillar squamous cell carcinoma (TSCC) is the most common type of oropharyngeal cancer, accounting for approximately 70–80% of all cancers of this anatomical region, representing a highly aggressive malignancy with early lymphatic dissemination [1, 2]. The incidence rate of TSCC has significantly increased from 0.35 to 1.5 cases per 100,000 individuals in the last three decades worldwide, especially amongst men and those aged 40–59 years [3]. In Korea, more than 2,500 new oropharyngeal cancer cases are annually diagnosed with an incidence rate of 5.7/100,000 and mortality rate of 2.0/100,000 individuals [4, 5]. Recently, human papillomavirus- (HPV-) related oropharyngeal squamous cell carcinomas (SCCs) have become an important subgroup of head and neck SCCs, and the tonsillar region is the most common location for HPV-associated head and neck SCCs. HPV-associated oropharyngeal SCCs are associated with an improved response to radiochemotherapy and have a better prognosis than HPV-negative tumors [6–8]. Molecular differences between HPV-positive and HPV-negative oropharyngeal SCCs have been identified and the two subgroups are considered to have differing pathogeneses and, consequently, altered therapeutic targets [8]. Several studies have shown that homeodomain-interacting protein kinase 2 (HIPK2) is an important tumor suppressor involved in HPV-associated uterine cervical and cutaneous carcinogenesis [9–11]. Therefore, we focused on aberrant HIPK2 overexpression in tonsil cancer and whether any identified correlations may explain the differences in prognosis or treatment outcome between HPV-positive and HPV-negative TSCC cases. HIPK2 is a nuclear serine/threonine kinase that acts as a corepressor for transcription factors and is one of the four multifunctional kinases of the HIPK family that are sensors for various extracellular stimuli. These kinases control key cellular functions such as signal transduction to downstream effectors that regulate apoptosis, embryonic development, DNA damage response, and cellular proliferation [12–14]; therefore, HIPK2 is involved in carcinogenesis.

The inactivation of the oncosuppressor protein p53 by the HPV E6 protein and the retinoblastoma protein (pRb) by the HPV E7 protein has been recognized as a pathogenic mechanism of HPV-associated tumor formation [15, 16]. HIPK2 is involved in apoptosis and is a central regulator of p53 [17]. Upon severe DNA damage, activated HIPK2 affects the upregulation of the proapoptotic function of p53 by specifically phosphorylating p53 at serine 46 (Ser46) and repressing its inhibitors, leading to apoptosis [12, 13, 18]. However, in HPV-infected cells, the binding of E6 to HIPK2 inhibits HIPK2-mediated p53 Ser46 phosphorylation by enforcing dissociation of the HIPK2/p53 complex [16], which prevents apoptosis and thus contributes to carcinogenesis [16]. The most notable properties of HIPK2 in tumors are that its inhibition or dysfunction leads to impairment of p53 function and the activation of oncogenic pathways that are important for tumor progression, angiogenesis, and resistance to chemotherapy or radiation therapy [17, 18], because HIPK2 is activated by several types of genotoxic damaging factors such as UV radiation, ionizing radiation, and antitumor drugs including cisplatin, adriamycin, and roscovitine [18–21]. Conversely, restoration of HIPK2 activity in tumor cells is effective for tumor regression. Because of its close relationship with HPV-associated oncogenic pathways and chemoradiation resistance, HIPK2 has recently attracted attention as a potential therapeutic target [17, 18].

As the efficacy of HIPK2 expression as a prognostic and predictive factor for HPV-positive head and neck cancers is yet to be demonstrated, the present study was performed to address this clinically relevant question in tonsil cancers, stratified by HPV status and postoperative radiation therapy.

## 2. Materials and Methods

### 2.1. Patients and Tissues

The present study was conducted using formalin-fixed, paraffin-embedded (FFPE) tissues obtained from 79 patients with primary TSCC who underwent surgery at the Ilsong Memorial Institute of Head and Neck Cancer, Kangdong Sacred Heart Hospital, between 1997 and 2010 [8]. The inclusion criteria included the following patients: (1) those who underwent primary resection, (2) those who received no prior treatment, and (3) those with available complete medical records, including pathologic slides and paraffin blocks of resected specimens. Normal tonsil samples (*n* = 20) as a control group were obtained from patients who underwent tonsillectomy owing to chronic follicular tonsillitis. Tonsil cancer cohorts in the present study had been enrolled in previously published studies [8, 22, 23]. The previous two studies included 47 patients of the present study cohort [22, 23]. The another previous study also included a total of 79 patients, which is the same patient cohort as the present study [24]. Normal tonsil specimens were not included in the previous studies. This retrospective study was undertaken after obtaining approval from the institutional review board of Kangdong Sacred Heart Hospital (IRB number 14-2-57).

Clinical information was analyzed using medical records and radiological study results. Smoking history was measured in pack-years, and patients were classified into 2 categories using 20 pack-years as the cut-off value, with heavy smoking defined as >20 pack-years [8, 23]. Similarly, alcohol consumption was divided into 2 categories using 14 drinks/week as the cut-off value, and heavy alcohol consumption was defined as >14 drinks/week [8, 23]. Surgical resection was followed by postoperative radiotherapy in 16 patients and chemotherapy or radiotherapy in 34 patients. Twenty-nine patients were treated with surgery alone. All patients underwent neck dissection on at least one side. Radiation doses ranged from 5040 cGy to 7200 cGy/36 fractions over a period of 8 weeks.

Histopathological characteristics were independently reviewed by two pathologists. Diagnosis and histological differentiation were evaluated according to the World Health Organization classification [25]. Staging was based on the American Joint Committee on Cancer staging system [24]. The tumor growth pattern at the invasive front was categorized as either pushing or infiltrative, with the former being a well-defined pushing margin with large tumor islands and the latter consisting of scattered small irregular cords or single tumor cells with a poorly defined infiltrating margin [8, 23].

### 2.2. Preparation of Tissue Microarray Blocks

For tissue microarray (TMA) construction, all H&E-stained slides were reviewed and representative areas were carefully selected. Each paraffin-embedded block relevant to hematoxylin and eosin slides was punched out by using a TMA manufacture tool (Quick-Ray™; Unitma, Seoul, South Korea). Three tissue cores (3 mm in diameter) were obtained separately from each tumor specimen. Tissue cores were arrayed in a recipient paraffin block according to the manufacturer's instructions. The 1 mm core of palatine tonsil next to the first tumor core was embedded to recognize the first case of this TMA block.

### 2.3. Immunohistochemistry

TMA tissue sections measuring 4 *μ*m in thickness were mounted on positively charged slides. These sections were deparaffinized with xylene and rehydrated through a gradient alcohol series. Staining was carried out according to the manufacturer's protocol on the BenchMark XT automated immunohistochemistry stainer (Ventana Medical Systems, Inc., Tucson, AZ, USA) using the* ultraView* DAB Kit (Ventana Medical Systems). The primary antibodies were HIPK2 (1 : 100; Abcam, Cambridge, UK) and p53 (1 : 500; Novocastra, Newcastle, UK) and were incubated as previously described [18].

Both the intensity of immunohistochemical staining and the proportion of stained tumor cells were evaluated and modified as previously described [10, 18]. The intensity of staining was divided into groups as follows: negative, weak, moderate, and strong. Only moderate or strong intensity staining was considered positive and counted in the percentage of positive cells. The staining proportion was rated according to the percentage of positive cells and expressed on a 5-point scale as follows: 0 for no stained cells; 1+ for staining of less than 10% of cells; 2+ for staining of 10 to 50% of cells; 3+ for staining of more than 50% of cells. Two pathologists (MJK and ESN) independently interpreted all the immunostained slides, and cases with discrepant scores were reevaluated to achieve a consensus score.

### 2.4. RNA Extraction and Quantitative Real-Time Reverse Transcription Polymerase Chain Reaction (RT-PCR)

*HIPK2* mRNA expression levels were measured using quantitative real-time RT-PCR by using TaqMan® Gene Expression Assays (Applied Biosystems Inc., Foster City, CA, USA; assay ID: HS00179759_m1 for HIPK2), as previously described [18]. The* GAPDH* gene (Applied Biosystems Inc.; assay ID: HS99999905_m1) was used as an endogenous control. Total RNA was isolated from 2 slices (10 *μ*m thickness) of FFPE tumor samples using the RNeasy Mini Kit (Qiagen, Hilden, Germany) according to the manufacturer's instructions [18]. Tumor cells were manually microdissected from paraffin sections of 10 *μ*m thickness under an inverted microscope to obtain a tumor cell population of >90%. RT-PCR was conducted using the High Capacity cDNA Reverse Transcription Kit (Applied Biosystems, cat. number 4368814) according to the manufacturer's instructions [18]. A total of 5 *μ*L of each cDNA sample was analyzed in triplicate using the ABI PRISM 7500HT Fast Real-Time PCR System (Applied Biosystems). C_T_, the fractional cycle number at which the amount of amplified target reached a fixed threshold, was determined, and the mRNA expression levels of each gene were measured using the 2^−ΔCt^  (ΔC_t_ = ΔC_target  gene_ − ΔC_GAPDH_) equation [18].

### 2.5. Peptide Nucleic Acid-Based Assay for HPV Detection

Genomic DNA was extracted from 10 *μ*m thick sections of 10% neutral FFPE tumor tissue blocks using the QIAamp DNA Mini Kit (Qiagen). The PANArray™ HPV chip test (PANAGENE, Daejeon, South Korea), a chip-based assay for detecting amplified HPV DNA of 32 genotypes (19 high- and 13 low-risk HPV types), was used for determination of HPV status according to the manufacturer's instructions [8, 26]. Detectable genotypes include HPV 16, HPV 18, HPV 26, HPV 31, HPV 33, HPV 35, HPV 39, HPV 45, HPV 51, HPV 52, HPV 53, HPV 56, HPV 58, HPV 59, HPV 66, HPV 68, HPV 69, HPV 70, and HPV 73 in the high-risk group and HPV 6, HPV 11, HPV 32, HPV 34, HPV 40, HPV 42, HPV 43, HPV 44, HPV 54, HPV 55, HPV 62, HPV 81, and HPV 83 in the low-risk group [8].

### 2.6. Statistical Analysis

Analyses of the correlations between the protein expression of HIPK2 and p53 and clinicopathological variables were carried out using the *χ*^2^ test or Fisher's exact test. Overall survival (OS) was defined as the interval from the first day of surgery until death or the end of follow-up. Disease-free survival (DFS) was defined as the interval from the first day of surgery until tumor progression, death, or the end of follow-up. Survival differences between individual groups were calculated using the Kaplan-Meier method with the log-rank test. We used the Cox proportional hazards model for the multivariate analysis of OS and DFS. OS and DFS rates were analyzed until January 2011. SPSS version 18 (SPSS Inc., Chicago, IL, USA) was used for all statistical analyses. A *P* value of <0.05 was considered statistically significant.

## 3. Results

### 3.1. Increased HIPK2 Protein Expression and* HIPK2* mRNA Expression Were Detected in Tonsil Cancers Compared to Normal Tonsillar Mucosa

**Figure 1:**
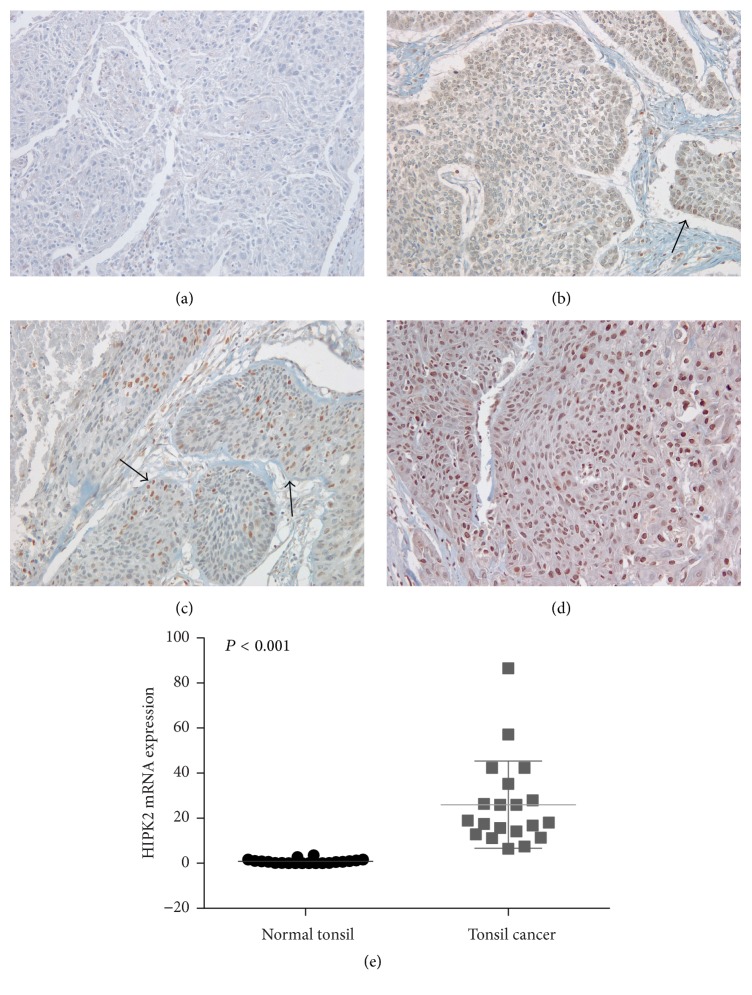
Representative photomicrographs of HIPK2 expression interpreted as 0 (a), 1+ (b), 2+ (c), and 3+ (d) in immunohistochemistry (*arrow*, positive nuclear staining). (e)* HIPK2* mRNA expression level in tonsillar squamous cell carcinoma is significantly increased compared to that of normal tonsil.

HIPK2 protein was weakly expressed in the nucleus and cytoplasm of the basal layer and was absent in the upper epithelial layers of normal tonsillar surface or crypt mucosa, whereas HIPK2 was diffusely expressed, mainly in the nuclei of tumor cells. Two cases showed both cytoplasmic and nuclear HIPK2 expression in the minority of well-differentiated, dyskeratotic tumor cells. Of the 79 TSCC tissue samples, HIPK2 overexpression was scored as 0 in 9 (11.4%), 1+ in 25 (31.6%), 2+ in 6 (7.6%), and 3+ in 39 (49.4%) cases (Figures 1(a)–1(d)). Thus, 45 cases (57.0%) with a score of 2+ or 3+ were considered HIPK2-positive. HIPK2 showed significantly different expression patterns between normal tonsillar mucosa and TSCC tissue (*P* = 0.036) (Table 1). While HIPK2 overexpression was observed in 57% of TSCC cases, its expression was significantly decreased in the normal tonsillar mucosa (27.8%, 5/18).

To elucidate the involvement of* HIPK2* in tonsil cancer, we analyzed* HIPK2* mRNA expression levels in 20 normal tonsil and 20 tonsil cancer FFPE samples. The* HIPK2* mRNA expression levels detected in TSCCs were significantly higher than those in the normal tonsil samples (*P* < 0.001; Figure 1(e)). The median values of* HIPK2* mRNA levels were 0.60 (0.02–3.56) in the normal tonsil and 18.47 (6.47–86.58) in TSCC tissue.

### 3.2. Clinicopathological Correlations with HIPK2 Expression

Patient characteristics and correlations with HIPK2 overexpression are summarized in Table 2. High alcohol consumption was more frequent in the HIPK2-negative group compared with the HIPK2-positive group (*P* = 0.002). HIPK2 overexpression was associated with poorly differentiated tumors (*P* = 0.043). HPV was detected in 28 (35.4%) of the 79 patients, where all cases were of the high-risk genotype 16. A total of 26.6% (21/79) of these HPV-positive samples were also p53-positive. However, HIPK2 expression was not associated with HPV positivity, smoking, or p53 expression (*P *= 1.000, *P* = 0.960, and *P* = 0.133, resp.).

### 3.3. Prognostic Significance of HIPK2 Overexpression

**Figure 2:**
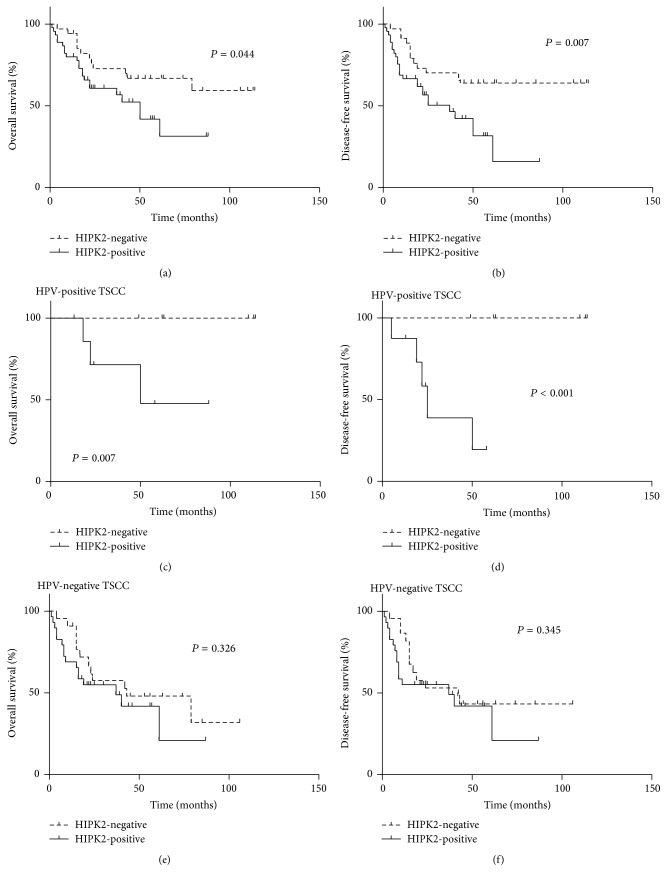
HIPK2 overexpression is associated with worse overall survival (a) and disease-free survival (b) in overall 79 patients with tonsillar squamous cell carcinoma. HIPK2 overexpression is associated with shorter overall survival (c) and disease-free survival (d) of the patients with HPV-positive tumors, respectively. However, HIPK2 overexpression has no prognostic impact on overall survival (e) or disease-free survival (f) of HPV-negative tumor patients.

Kaplan-Meier survival analysis showed that HIPK2-positive patients had shorter OS and DFS than HIPK2-negative patients (OS, mean 47 months versus 80 months, *P* = 0.044; DFS, 37 months versus 79 months, *P* = 0.007, resp.; Figures 2(a) and 2(b)). Following multivariate analysis with variables including HPV status, age, smoking, pT category, histologic differentiation, contralateral cervical nodal status, and HIPK2 expression status, HIPK2 overexpression was identified as an independent prognostic factor for patient OS and DFS (*P* = 0.007, hazard ratio [HR] = 2.958, 95% confidence interval [95% CI]: 1.353–6.465; *P* = 0.004, HR = 3.004, 95% CI: 1.415–6.377, resp.; Table 3).

### 3.4. Prognostic Associations of HIPK2 Overexpression with HPV Infection and Postoperative Radiation Therapy

We further analyzed the prognostic value of HIPK2 expression for OS and DFS according to HPV status. HIPK2 overexpression was strongly correlated with poorer OS and DFS in HPV-positive TSCC cases (*P* = 0.007 and *P* < 0.001, resp.; Figures 2(c) and 2(d)). However, the correlations between HIPK2 overexpression and OS or DFS in patients with HPV-negative tonsil cancer were not statistically significant (*P* = 0.326 and *P* = 0.345, resp.; Figures 2(e) and 2(f)).

**Figure 3:**
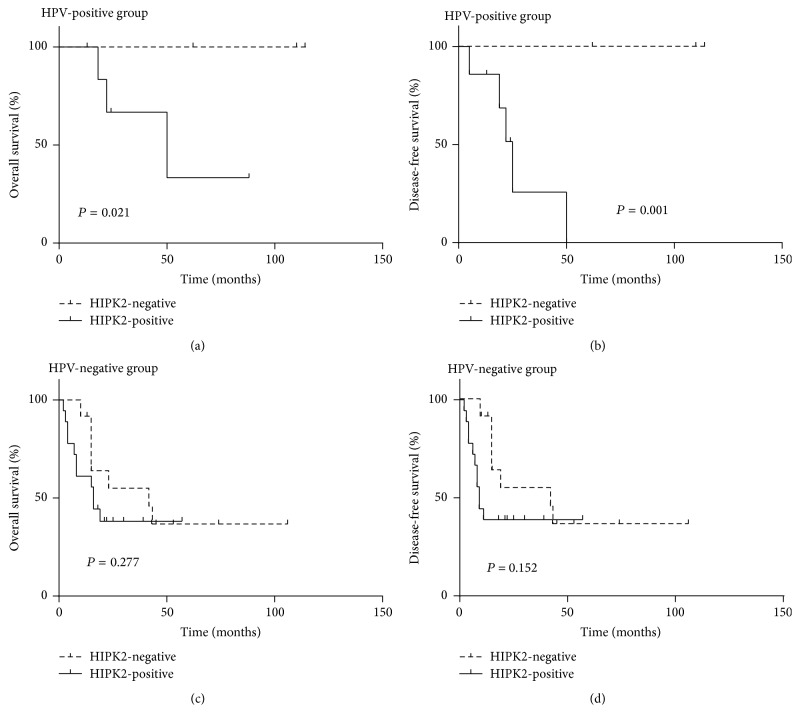
Overall survival (a, c) and disease-free survival (b, d) by HIPK2 expression status in the resection group receiving postoperative radiation therapy according to HPV status. HIPK2 overexpression is strongly correlated with decreased overall survival (a) and disease-free survival (b) in the patients with HPV-positive tonsil cancers. However, HIPK2 expression was not associated with overall survival (c) or disease-free survival (d) in the patients with HPV-negative tumors.

We also investigated the prognostic differences associated with HIPK2 overexpression according to HPV status in patients with TSCC who received postoperative radiation therapy. The HIPK2 overexpression in HPV-positive tumors was strongly correlated with decreased OS and DFS in the patients who received postoperative radiation therapy (*P* = 0.021 and *P* = 0.001, resp.; Figures 3(a) and 3(b)). In patients with HPV-positive tumors, those with HIPK2 overexpression had significantly shorter OS and DFS than those with no HIPK2 expression (OS, median 24 months versus 110 months; DFS, median 18 months versus 110 months, resp.).

Multivariate analyses including HIPK2 overexpression, HPV infection, age, pT category, and contralateral nodal metastasis revealed that HIPK2 overexpression was an independent prognostic factor for DFS (*P* = 0.027, HR = 3.049, 95% CI: 1.133–8.206) in the radiation therapy group. On the other hand, HPV was an independent prognostic factor for the OS of the patients receiving radiation therapy (*P* = 0.010, HR = 0.220, 95% CI: 0.069–0.694). However, there was no statistically significant difference between OS or DFS and HIPK2 overexpression in the patients with HPV-negative tumors who received radiation therapy (*P* = 0.277 and *P* = 0.152, resp.; Table 4).

## 4. Discussion

In recent years, there has been a growing interest in increasing the risk-benefit ratio in the clinical outcome of HPV-positive locally advanced oropharyngeal cancers. In the present study, we first investigated HIPK2 overexpression in patients with TSCC as a predictor of clinical outcomes related to HPV. HIPK2 overexpression was associated with poorly differentiated tonsil cancer, which is an unfavorable histological factor in patients with TSCC. HIPK2 overexpression was identified as an independent negative prognostic factor and was associated with decreased OS and DFS in patients with tonsil cancer, specifically in those with HPV-positive TSCC. However, these prognostic correlations associated with HIPK2 overexpression were not identified in patients with HPV-negative tonsil cancer. We also found that HIPK2 overexpression was associated with low alcohol consumption in patients with TSCC. This result appears to be partially comparable with those of previous studies referring to smoking and alcohol consumption as risk factors for the development of HPV-negative TSCC [27]. As the tonsillar region is the most common location for HPV-associated head and neck SCCs, HIPK2 overexpression in HPV-positive tonsil cancers may be a poor prognostic indicator in a subset of TSCCs, which may develop in individuals who do not consume alcohol.

HIPK2 was highly expressed in 57% of TSCCs, and HIPK2 protein expression and* HIPK2* mRNA levels were significantly higher in TSCCs than in normal tonsil tissues. These findings may suggest that HIPK2 overexpression is relatively common in TSCC and may be involved in the tumor development of tonsil cancers. Concordantly, D'Orazi et al. [28] reported that* HIPK2* mRNA levels in colorectal cancers are higher than those in normal colon cells [18]. HIPK2 is overexpressed in TSCCs, which differs from previously reported results for other human malignancies [18, 28, 29], with the exception of uterine cervical cancers where HPV infection is prevalent [11]. HIPK2 protein expression in cervical cancer gradually increases along with disease progression in intraepithelial cervical neoplasia, carcinoma in situ, and invasive cervical cancer [11, 18], implicating the contribution of HIPK2 to the tumor development and progression of uterine cervical neoplasms. HIPK2 is more highly expressed in HPV-associated cervical SCCs (72.7%) than in cutaneous SCCs (14.3%) without HPV infection [10, 18]. In contrast, the loss of HIPK2 protein expression has been initially described with aggressive behavior and tumor progression in colorectal and thyroid cancers [28, 29]. HIPK2 is undetectable in 91.7% of papillary thyroid carcinomas and in 60.0% of follicular thyroid carcinomas, compared with its overexpression in benign nodular hyperplasia [18, 29]. Soubeyran et al. [30] have shown that the increased expression of the HIPK2 protein in colorectal tumor cells compared with paired normal tissue cells has a strong impact on improved survival of patients with colorectal cancer. The paradoxical biological behaviors of HIPK2 overexpression in HPV-associated carcinomas are closely linked with the oncogene E6, encoded by high-risk HPV, and HIPK2 [16]. E6 interacts with HIPK2, inhibiting HIPK2-mediated p53 Ser46 phosphorylation by enforcing dissociation of the HIPK2/p53 complex [16]. Thus, HPV interferes with the cellular apoptosis response and contributes to the development of SCCs [16].

HIPK2 may be an attractive biomarker and a therapeutic target [31], particularly in patients with cancer and HPV infection. The maintenance of intact HIPK2 function is important in anticancer therapy, and its functional inhibition appears to be associated with chemoresistance and radioresistance [17, 18, 32]. Aberrant overexpression of HIPK2 protein may occur owing to an underlying abnormal* HIPK2* gene, leading to the expression of mutated protein [11]. Here, we demonstrated that HIPK2 overexpression was a negative independent prognostic factor for DFS in the postoperative radiotherapy group. HIPK2 expression can be readily assessed using immunohistochemistry in routinely processed tissue samples. As the majority of patients with tonsillar cancer undergo radiation therapy with or without chemotherapy as a primary treatment, HIPK2 expression could represent a promising tissue marker for the prognosis of patients with TSCC who receive postoperative treatment.

The retrospective procurement of archival samples and the limited number of patients may be limitations of the current study. Nevertheless, certain trends emerged from the results. HIPK2 overexpression may predict a poor prognosis and a high risk of recurrence in patients with TSCC, especially those with HPV-positive tonsil cancers and those receiving postoperative radiation therapy, implying that HIPK2 could be a potential prognostic and predictive biomarker for tonsil cancer.

## Figures and Tables

**Table 1:** Correlation of HIPK2 expression between normal tonsillar mucosa and tonsillar squamous cell carcinoma (TSCC).

Variable	HIPK2		*P*
	Positive	Negative	
Normal mucosa (*n* = 18) (%)	5 (27.8)	13 (72.2)	0.036^*∗*^
TSCC (*n* = 79) (%)	45 (57.0)	34 (43.0)	

^*∗*^Statistically significant, *P* value < 0.05.

**Table 2:** Association between HIPK2 expression and tonsil cancer patient characteristics.

Variable	Total	HIPK2		*P*
		Positive	Negative	
	*N* = 79 (%)	*n* = 45 (%)	*n* = 34 (%)	
Gender				0.406
Male	68 (86.1)	40 (88.9)	28 (82.4)	
Female	11 (13.9)	5 (11.1)	6 (17.6)	
Age (y)				0.079
≤60	56 (70.9)	28 (62.2)	28 (82.4)	
>60	23 (29.1)	17 (37.8)	6 (17.6)	
Smoking (pack-years)				0.960
<20	23 (29.1)	13 (28.9)	10 (29.4)	
≥20	56 (70.9)	32 (71.1)	24 (70.6)	
Alcohol (drinks/week)				0.002^*∗*^
<14	32 (40.5)	25 (55.6)	7 (20.6)	
≥14	47 (59.5)	20 (44.4)	27 (79.4)	
T classification				0.570
pT1-pT2	47 (59.5)	28 (62.2)	19 (55.9)	
pT3-pT4	32 (40.5)	17 (37.8)	15 (44.1)	
AJCC stage				0.861
I-II	11 (13.9)	6 (13.3)	5 (14.7)	
III-IV	68 (86.1)	39 (86.7)	29 (85.3)	
Depth of invasion				0.105
<2 cm	61 (77.2)	38 (84.4)	23 (67.6)	
≥2 cm	18 (22.8)	7 (15.6)	11 (32.4)	
Tumor differentiation				0.043^*∗*^
W/M	53 (67.1)	26 (57.8)	27 (79.4)	
Poorly	26 (32.9)	19 (42.2)	7 (20.6)	
Tumor-stromal border				0.495
Pushing	42 (53.2)	22 (48.9)	20 (58.8)	
Infiltrative	37 (46.8)	23 (51.1)	14 (41.2)	
Lymphatic invasion				0.095
Present	63 (79.7)	39 (86.7)	24 (70.6)	
Absent	16 (20.3)	6 (13.3)	10 (29.4)	
ILN status				0.375
Metastasis	66 (83.5)	36 (80.0)	30 (88.2)	
No metastasis	13 (16.5)	9 (20.0)	4 (11.8)	
CLN status				0.254
Metastasis	14 (17.7)	6 (13.3)	8 (23.5)	
No metastasis	65 (82.3)	39 (86.7)	26 (76.5)	
HPV status				1.000
Positive	28 (35.4)	16 (35.6)	12 (35.3)	
Negative	51 (64.6)	29 (64.4)	22 (64.7)	
p53				0.133
Positive	21 (26.6)	15 (33.3)	6 (17.6)	
Negative	58 (73.4)	30 (66.7)	28 (82.4)	

AJCC: American Joint Committee on Cancer; W: well differentiated; M: moderately differentiated; ILN: ipsilateral cervical lymph node; CLN: contralateral cervical lymph node metastasis; HPV: human papillomavirus. ^*∗*^Statistically significant, *P* value < 0.05.

**Table 3:** Multivariate analysis of overall survival and disease-free survival in TSCC patients.

	Overall survival	*P*	Disease-free survival	*P*
	Hazard ratio (95% CI)		Hazard ratio (95% CI)	
HIPK2 expression		0.007^*∗*^		0.004^*∗*^
Negative versus positive	2.958 (1.353–6.465)		3.004 (1.415–6.377)	
HPV		0.013^*∗*^		0.026^*∗*^
Negative versus positive	0.324 (0.133–0.787)		0.358 (0.145–0.884)	
Age (years)		0.034^*∗*^		0.047^*∗*^
≤60 versus >60	2.383 (1.068–5.314)		2.295 (1.011–5.206)	
Smoking (pack-years)		—		0.331
<20 versus ≥20	—		1.573 (0.631–3.923)	
pT category		0.003^*∗*^		0.007^*∗*^
pT1 and pT2 versus pT3 and pT4	3.214 (1.478–6.989)		2.987 (1.349–6.613)	
Differentiation		0.894		—
W/M versus poorly	1.049 (0.521–2.110)		—	
CLN		0.021^*∗*^		0.023^*∗*^
No versus yes	2.689 (1.164–6.214)		2.571 (1.136–5.820)	

TSCC: tonsillar squamous cell carcinoma; CI: confidence interval; HPV: human papillomavirus; W: well differentiated; M: moderately differentiated; CLN: contralateral cervical lymph node metastasis. ^*∗*^Statistically significant, *P* value < 0.05.

**Table 4:** Univariate and multivariate analyses in the patients with postoperative radiation therapy affecting the overall and disease-free survival rates.

	Overall survival			Disease-free survival		
	Univariate	Multivariate	*P*	Univariate	Multivariate	*P*
	*P*	HR (95% CI)		*P*	HR (95% CI)	
HPV	0.008^*∗*^		0.010^*∗*^	0.148		0.227
Negative versus positive		0.220 (0.069–0.694)			0.560 (0.218–1.436)	
HIPK2 expression	0.103		0.062	0.007^*∗*^		0.027^*∗*^
Negative versus positive		2.775 (0.951–8.095)			3.049 (1.133–8.206)	
Age (years)	0.003^*∗*^		0.009^*∗*^	0.006^*∗*^		0.004^*∗*^
≤60 versus >60		4.362 (1.438–13.234)			4.698 (1.646–13.411)	
pT category	0.023^*∗*^		0.008^*∗*^	0.045^*∗*^		0.026^*∗*^
pT1 and pT2 versus pT3 and pT4		3.925 (1.422–10.832)			2.883 (1.135–7.327)	
CLN	0.001^*∗*^		0.005^*∗*^	0.018^*∗*^		0.118
No versus yes		3.645 (1.469–9.044)			1.961 (0.843–4.563)	

HR: hazard ratio; CI: confidence interval; HPV: human papillomavirus; CLN: contralateral cervical lymph node metastasis. ^*∗*^Statistically significant, *P* value < 0.05.
